# Advances in machine learning for ABCA4-related retinopathy: segmentation and phenotyping

**DOI:** 10.1007/s10792-025-03690-4

**Published:** 2025-07-23

**Authors:** Yousif J. Shwetar, Brian P. Brooks, Brett G. Jeffrey, Benjamin D. Solomon, Melissa A. Haendel

**Affiliations:** 1 Joint Department of Biomedical Engineering, University of North Carolina and North Carolina State University, Chapel Hill, NC USA; 2 Ophthalmic Genetics and Visual Function Branch, National Eye Institute, NIH, Bethesda, MD USA; 3 Medical Genetics Branch, National Human Genome Research Institute, NIH, Bethesda, MD USA; 4 Department of Genetics, University of North Carolina, Chapel Hill, NC USA

**Keywords:** Stargardt disease, ABCA4-related retinopathy, Machine learning, Retinal image segmentation, Phenotyping

## Abstract

**Purpose:**

Stargardt disease, also called ABCA4-related retinopathy (ABCA4R), is the most common form of juvenile-onset macular dystrophy and yet lacks an FDA approved treatment. Substantial progress has been made through landmark studies like that of the Progression of Atrophy Secondary to Stargardt Disease (ProgStar), but tasks like image segmentation and phenotyping still pose major challenges in terms of monitoring disease progression and categorizing patient subgroups. Furthermore, these methods are subjective and laborious. Recent advancements in machine learning (ML) and deep learning show considerable promise in automating these processes.

**Methods:**

This scoping review explores ML applications in ABCA4R, with a focus on segmentation and phenotyping. Following the Preferred Reporting Items for Systematic Reviews and Meta-Analysis (PRISMA) methodology, 15 articles were selected from 264, with 12 focused on the task of segmenting atrophic lesions, retinal flecks, retinal layer boundaries, or en-face imaging. Three studies addressed phenotyping based on electroretinography (ERG), visual acuity, and microperimetry.

**Results:**

Several effective approaches were implemented in these studies, including ensemble modeling, self-attention mechanisms, soft-label approaches, and dynamic frameworks that consider extent of tissue damage. Excellent model performance includes segmentation DICE performances of 0.99 and ERG phenotyping accuracies 90% and greater. Smaller datasets and variable presentations present as significant challenges, while advanced methods like Monte Carlo dropout and active learning improve pipeline efficiency and performance.

**Conclusion:**

ML techniques are well on their way to automate key steps in ABCA4R evaluation with excellent performance. These emerging methods have the potential to expedite therapeutic innovation and enhance our understanding of ABCA4R.

## Introduction

**Fig. 1:**
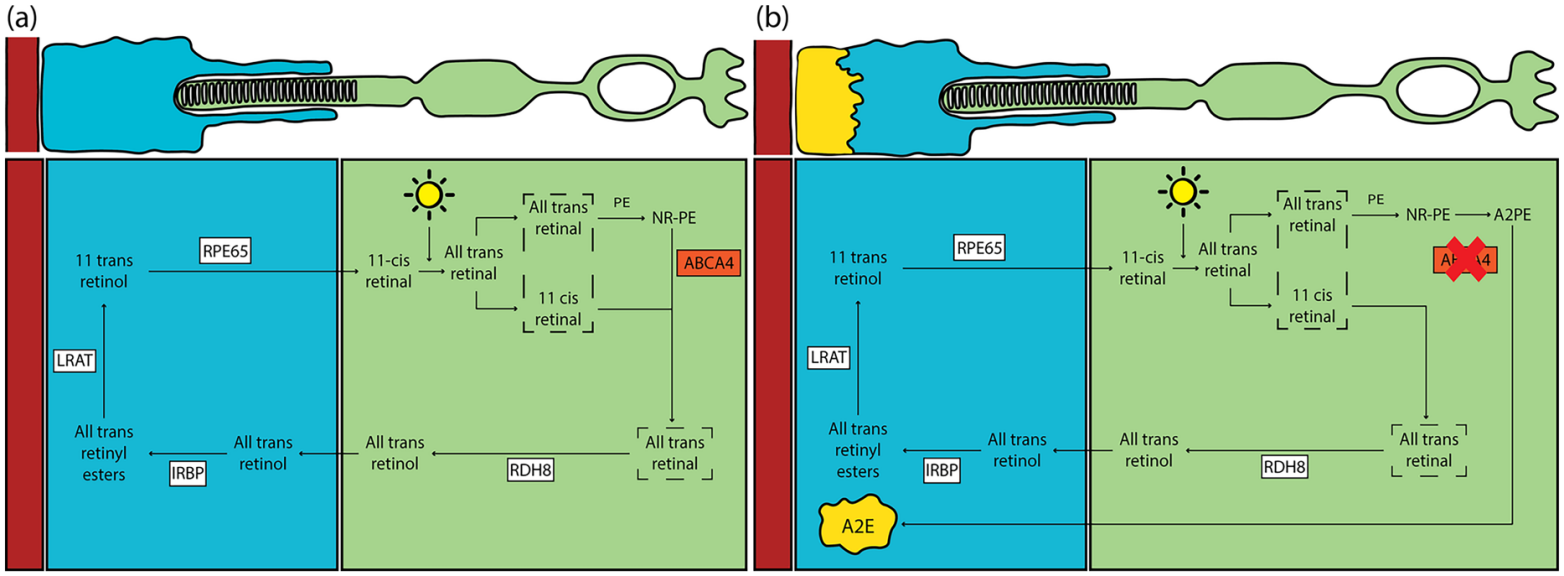
ABCA4 Pathway in Healthy vs ABCA4R. **a** Healthy Retina: Light triggers the conversion of 11-cis retinal to all-trans retinal within photoreceptor discs. All-trans retinal then bind to Phosphatidylethanolamine (PE) forming N-retinylidene-Phosphatidylethanolamine (NR-PE), which is then exported via the functional ABCA4 transporter. All-trans retinal is subsequently reduced to all-trans retinol by Retinol Dehydrogenase 8 (RDH8), moved to the retinal pigment epithelium (RPE) by Interphotoreceptor Retinoid Binding Protein (IRBP), and then esterified by Lecithin Retinol Acyltransferase (LRAT). 11-cis retinol is then regenerated by Retinal Pigment Epithelium-specific Protein 65 kDa (RPE65), completing the retinoid cycle. **b** ABCA4R: Pathogenic ABCA4 variants disrupt the export of NR-PE, causing the buildup of A2-Phosphatidylethanolamine (A2PE) and its toxic derivative, N-retinylidene-N-retinylethanolamine (A2E). These yellow deposits accumulate in the RPE, ultimately damaging the RPE and overlying photoreceptors, leading to the characteristic atrophy observed in ABCA4-related retinopathy

Stargardt disease (STGD1, OMIM #248200, MONDO:0019353) was first described by Physician Karl Stargardt in 1909 in seven individuals who presented with macular dystrophy surrounded by pisciform flecks [1]. Studies later identified autosomal recessive variants in the *ABCA4* gene resulting in a loss of function of the ABCA4 lipid transporter, essential for the phototransduction cascade in photoreceptors [2, 3] and retinal pigment epithelium (RPE) [3, 4]. A detailed overview of the phototransduction cascade in healthy individuals and those with Stargardt disease can be found in (Fig. 1). Variants in the *ABCA4* gene are now understood as a spectrum of phenotypes–macular dystrophy/atrophy, cone-rod degeneration, rod-cone degeneration—collectively termed ABCA4-Related Retinopathy (ABCA4R), affecting approximately 1 in 8000–10,000 individuals worldwide [5–7].

**Fig. 2:**
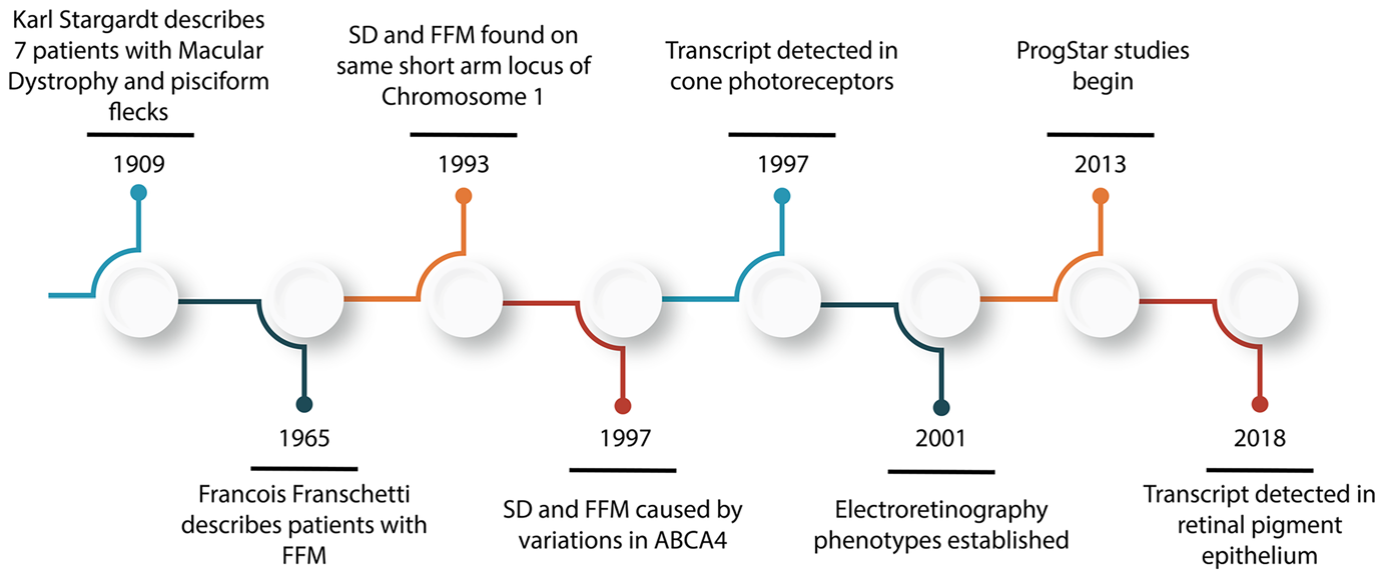
Timeline of Major Milestones in ABCA4R Research, from Karl Stargardt’s first description in 1909 to the emergence of large-scale studies such as ProgStar and transcript detection in the retinal pigment epithelium. SD: Stargardt disease, FFM: Fundus Flavimaculatus

Despite being the leading cause of juvenile macular dystrophy (JMD) [8, 9], and one of the most common inherited retinal dystrophies (IRD) [10], there are no approved treatments by the FDA for ABCA4R [11]. Several clinical trials have been completed or are underway to address ABCA4R’s functional loss using a spectrum of strategies ranging from small molecules to gene replacement [12–14]. However due to the rarity of this condition, information about its natural history has historically been limited [15–18]. More recently, several groups have reported on the natural history of ABCA4R progression using a variety of clinical and imaging modalities, all with the purpose of identifying potential outcome variables for use in human clinical trials [19]. The largest of these, ProgStar, was launched by the Foundation for Fighting Blindness in 2013, publishing multiple studies to this purpose [9, 20–36]. An overview of key milestones in history of Stargardt disease discovery are outlined in (Fig. 2).

While these large-scale studies have significantly advanced the understanding of ABCA4R, challenges remain in efficiently collecting and analyzing patient data. For instance, segmenting (the process of locating and partitioning images into meaningful regions) flecks in fundus autofluorescence (FAF) or retinal layers in spectral domain optical coherence tomography (SD-OCT) are time-consuming when done manually, and require specialized training [37, 38]. Similarly, effective phenotyping of patients using electroretinography (ERG) recordings or microperimetry demands similar expertise [39, 40]. Atrophic lesions represent degeneration of macular photoreceptors and RPE, resulting in central hypoautofluorescence and retinal layer thinning in FAF and SD-OCT imaging, respectively. Flecks represent the accumulation of lipofuscin byproducts, and present throughout the retina in both FAF and SD-OCT imaging. Retinal layer segmentation is only through SD-OCT imaging, which can demonstrate both atrophic lesion progression, lipofuscin buildup, and overall retinal health. Automated segmentation can significantly reduce time and burden on clinicians, allowing more time to conduct confirmatory testing or patient consultations. The same is seen in the task of phenotyping, which involves the use of ML models to stratify patients based on severity, using ERG findings, microperimetry, and potential proxy measures of visual function, streamlining the interpretation of disease status and progression. Some of these tasks involve a degree of subjectivity while others have significant challenges with test/retest reliability. Most of all, reliable prognostication of future atrophy and other sequelae remains a significant challenge, with limited information that can answer these questions at presentation. Automation methods that are both efficient and accurate could mitigate these limitations and support clinical trials in recruiting as well as following larger cohorts. Synthesizing data from these cohorts could enable the use of proxy measures, conserving resources in both primary care and clinical research settings.

Recent advances in artificial intelligence (AI) are positioned to address many of the aforementioned limitations [41, 42]. AI refers to computational systems that can perform tasks typically requiring human intelligence, including many types of pattern recognition, decision making, and outcome prediction. A type of AI, Machine Learning (ML), focuses on algorithms that learn patterns from data to make predictions or decisions, without being directly programmed to do so. ML models can be trained with labels such as a diagnosis (supervised learning), uncover hidden structures in unlabeled data (unsupervised learning), or iteratively improve performance based on feedback from a user such as a clinician (reinforcement learning). Deep Learning (DL) is a sub-branch of ML that uses artificial neural networks with many hidden layers, to automatically extract high-level features from raw data, such as medical images or written clinical documentation. Large datasets are valuable in these endeavors in order to avoid overfitting as well as ensure generalizability. Although conditions like ABCA4R are rare, they have benefited from structured initiatives like ProgStar and longitudinal tracking by groups at the National Eye Institute (NEI). With promising therapeutics on the horizon, there is an urgent need for automated and effective methods to quantitatively track disease progression. By using advanced learning tools on these large and diverse datasets, researchers are able to derive consistent and objective metrics, reducing variability as well as allowing comparisons across large datasets. For phenotyping tasks, ML tools can categorize patients based on psychophysical and/or electrophysiological measures, improving stratification in clinical trials. Alternatively, DL models can segment images quickly, do so with high precision, and even predict future atrophy progression. These methods empower clinical trials with the ability to recruit larger cohorts, generate more conclusive and generalizable findings, and reduce personnel demands for manual tasks such as segmentation. By addressing current limitations, these AI-driven approaches not only enhance prognostication but also accelerate the discovery and validation of therapeutic interventions.

Here, we present a scoping review that assesses the current status of ML applications in ABCA4R that focus on automating phenotyping and segmentation. This scoping review documents current approaches that are most promising, as well as tools that ameliorate ML challenges in ABCA4R such as those related to limited dataset sizes. These insights will empower clinicians and researchers to contribute robust and effective learning methods for the much needed care and discovery that would benefit the field and, ultimately, affected individuals.

## Materials and methods

**Fig. 3:**
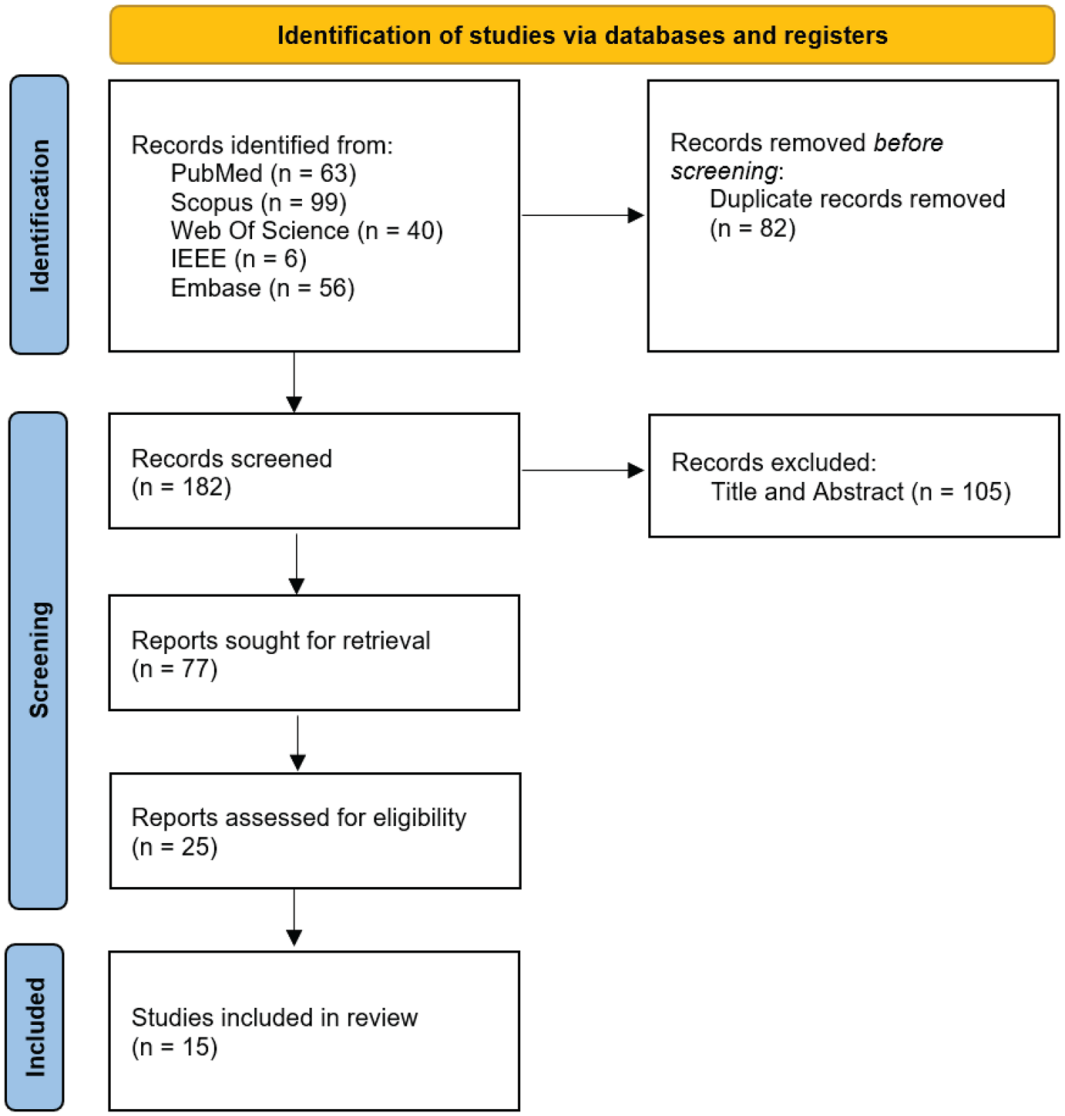
PRISMA Flow Diagram. Illustration of the systematic search and selection process. Each step shows the number of records identified, screened, and excluded, leading to the final 15 studies included in this scoping review. IEEE: Institute of Electrical and Electronics Engineers

A scoping review was performed to assess the current status of ML for segmentation and phenotyping in ABCA4R. The study was conducted following the Preferred Reporting Items for Systematic Reviews and Meta-Analyses (PRISMA) methodology [43]. Several electronic databases were used to identify studies that met the inclusion and exclusion criteria for this scoping review including PubMed, Scopus, Web of Science, IEEE Xplore, and Embase. A PRISMA flow diagram in (Fig. 3) presents this iterative process. The following terms were used in the search to capture a broad range of relevant machine learning technologies: “Artificial Intelligence,” “Machine Learning,” “Deep Learning,” “Neural Networks,” “Algorithms,” and “Image Processing, Computer-Assisted.” These terms were selected to ensure comprehensive coverage of studies applying machine learning approaches, from diagnostic imaging analysis to predictive modeling and disease progression tracking.

In parallel, the following disease-specific terms were applied to focus the search on ABCA4-related retinopathy: “Stargardt disease,” “Stargardt disease 1,” “STGD1,” “ABCA4-related retinopathy,” and “Juvenile Macular Degeneration.” These terms were chosen to account for the diverse terminology used in the literature, ensuring that all relevant studies on ABCA4-associated retinal conditions were included. Studies were included if they focused on ABCA4R or one of its child terms as defined by the MONDO Disease Ontology[44], implement at least one of the aforementioned ML approaches (supervised, unsupervised, or reinforcement learning), and address a key challenge in ML such as image segmentation or phenotyping. There are three segmentation tasks—atrophic lesions, flecks, and retinal layers. Studies were excluded if they did not meet the inclusion criteria, were not in English, or were classified as a review or survey. For each article included in this review, pertinent information was extracted including imaging modality, dataset size, ML problem, models used, and the best performance.

Two reviewers independently assessed each study for eligibility based on predefined criteria, with a third reviewer available to resolve any disagreements. This structured approach facilitated a thorough and systematic evaluation of the available literature. The review process concluded on April 4th, 2025.

## Results

In total, 266 references were extracted, with 82 detected as duplicates by automation software. Of the 184 screened, 107 were excluded as they failed to meet one of the pre-specified criteria: (i) the study did *not* focus on ABCA4R or a related child term, (ii) it did *not* implement a ML approach (iii) it did *not* address a key ML challenge relevant to this review (iv) it was published in a non-English language, or (v) it was a not an original research article. This left 77 reports sought for retrieval. A final 25 reports were assessed for eligibility, resulting in the removal of 10 reports due to a lack of a learning method, or classifier model only.

Table 1 depicts the final 15 included articles [45–59] in this review with pertinent information regarding modality, dataset size, purpose, ML information, and results [60]. All included articles are relatively recent, with the oldest article from 2018. The year 2022 had the most references at four, with 2020 and 2021 with three each. Of the 15 studies, two also investigated Age Related Macular Degeneration (AMD) simultaneously. Furthermore, Wang and colleagues [45] specifically collected JMD, being the only study to do so. For modality used, OCT was the most used modality across all studies at 9 of 15 studies including it, followed closely by 7 of 15 studies using FAF, three studies implementing ERG, two with Best-Corrected Visual Acuity (BCVA) and a single study collecting microperimetry (MP). Twelve of 15 studies utilized imaging alone, with 5 studies using OCT only, 5 using FAF only, and two using both FAF and OCT. One study utilized only ERG, while the remaining two used a combination of BCVA, ERG, MP, and OCT.

**Table 1:** Details of the final retrieved studies

AI problem	Study	Modality	Dataset	Model(s)	Best performance
Segmentation (Atrophic Lesion)	[45]	FAF	100 FAF Eyes	U-Net	DICE = 0.87 ± 0.13
	[46]		206 FAF Eyes 127 Patients	ResNet U-Net	DICE = 0.79 ± 0.03
	[47]		193 FAF Eyes	ResNet	DICE = 0.90
Segmentation (Flecks)	[48]	FAF	47 FAF Eyes 27 Patients	ResNet	DICE = 0.33—0.80
	[49]		81 FAF Eyes 41 Patients		DICE = 0.53 ± 0.13
Segmentation (Retinal Layers)	[50]	OCT	44 OCT Eyes 22 Patients	U-Net	ILM: MAE = 0.23 (0.30) RPE: MAE = 1.12 (1.41)
	[51]		197 OCT Eyes	U-Net	MAE Range = 0.77 (2.15)–4.73 (6.00)
	[52]		18 Patients	Monte-Carlo, U-Net	ILM: MAE = 0.51 (1.73) RPE: MAE = 1.66 (2.24)
	[53]		259 Patients	Faster R-CNN, DeepLabv3, U-Net, ReLayNet	DICE = 0.99
Segmentation (Mixed)	[54]	OCT	132 Eyes 66 Patients	DeepLabv3	DICE = 0.97
	[55]	FAF, OCT	264 FAF Eyes 264 OCT Eyes	Ensemble (Feature maps): U-Net	DICE = 0.830
	[56]		141 FAF Eyes 71 OCT Eyes	LSTM, U-Net	DICE = 0.922
Phenotyping	[57]	BCVA, ERG, OCT	311 Eyes 156 Patients	Ensemble Classification, Ensemble Regression	ERG Class Acc = 99.53% BCVA Class Acc = 89.10%-93.68%
	[58]	BCVA, ERG, MP, OCT	267 Eyes 134 Patients	RF Regression	MAE [95% CI] = 3.89 dB [3.11 dB—3.91 dB]
	[59]	ERG	1189 ERG Eyes 597 Patients	Ensemble Classification	ERG Class Acc = 91.8%

Summary of the 15 articles included in this scoping review of ML-based segmentation and phenotyping approaches in ABCA4-related retinopathy. Columns detail imaging modality, dataset size, specific ML task, type of model(s), and a key performance metric or outcome

Segmentation was the most common DL task, with 12 of 15 of the studies conducting this as their only task, using either FAF, OCT, or both. Specifically Retinal Layer segmentation consisted of four of these using only OCT, while Atrophic Lesions and Flecks using FAF consisted of three and two respectively. Three studies performed retinal layer segmentation to achieve en-face projections, termed here as ‘mixed methods’, with one using only OCT, and the latter two using both OCT and FAF. All phenotyping studies included a collection of features in their analysis like objective measures from imaging (retinal layer thickness from OCT scans), electrophysiological recordings (ERG), demographic information, physical eye characteristics (pupil diameter), among others.

Dataset categorization varied across studies, with only four of 15 studies including healthy subjects. Both studies by Muller [57, 58] included healthy subjects in their predictions while Alone-Canerio et al. [51] and Pfau et al. [54] included healthy subjects to aid in the task of segmenting all retinal layers. Three studies reported less than 100 eyes of data in developing their model, while 10 studies were within the range of 100–311 eyes of data. Glinton [59] boasted the largest number of eye samples with 1189 eyes with ERG recordings.

Due to differences in the ML tasks studied in the papers, there were inherent differences in what metrics were used to assess performance. A concerted effort was implemented here to report metrics that best describe overall model performance, while also considering which metrics were widely shared across studies. For example, DICE is reported in (Table 1) instead of accuracy as the former is a better quantitative measure of segmentation performance. Khateri and colleagues was the only study to report DICE for retinal layer segmentation, while the remaining three studies reported differences in segmenting paths as mean absolute error (MAE). ERG classification performance would be better described with sensitivity and specificity, but only Glinton et al. [59] reported this, so we reported classification accuracy to aid direct comparisons.

## Segmentation

Twelve articles focused on the task of Segmentation, which can be divided into four sub-categories: Atrophic Lesion, Flecks, Retinal Layers, and Mixed Methods.

Three articles focused on the segmentation task of Atrophic Lesions [45–47]. Wang et al. [45] used AMD subjects for model development and tested on JMD cases with 100 FAF images, while their follow-up study [46] expanded to 206 FAF images. Both studies utilized a U-Net model, with the latter adding self-attention mechanisms for both baseline segmentation and future atrophy prediction. Performance comparison between the original and self-attended model showed DICE values of 0.65 ± 0.03 and 0.79 ± 0.03 respectively. Alternatively Zhao and colleagues [47] segmented atrophic lesions based on autofluorescence lesion types, for regions with definitely decreased autofluorescence (DDAF, AF darkness is 90% that of the optic disc), questionably decreased autofluorescence (QDAF, AF darkness is 50–90% that of the optic disc) and other lesion types in 193 eyes with Stargardt disease. This group utilized a U-Net with a ResNet-50 encoder (ResNet-U-Net) architecture, reporting a best DICE of 0.90 for DDAF lesions and 0.78 across other lesion classes.

Two studies addressed fleck segmentation [48, 49]. Charng and colleagues [48] implemented a 12-parameter transformation matrix, iterative closest point (ICP) algorithm, and contrast limited adaptive histogram equalization (CLAHE). Progression was tracked using ETDRS visual angles (0–10, 10–20, 20–30), while a study by Sabharwal et al. [49] measured fleck characteristics including size (mm^2^), circularity, distance to fovea (mm^2^), and distance to nearest fleck (µm^2^). Both utilized a ResNet-U-Net model with residual blocks replacing standard convolution and max pooling in the encoder. The latter [49] also applied a k-means clustering to these fleck characteristics. The baseline analysis for these studies included 47 FAF images (27 patients) [48] and 81 FAF images (41 patients) [49], achieving DICE ranges of 0.33–0.80 and mean DICE of 0.53 respectively. Longitudinal analysis by Charngs group [48] included 82 FAF images from 6 patients over 12 months.

Four studies focused on retinal layer segmentation [50–53]. Alonso-caneiro and Kugleman et al. in two different studies [50, 52] focused on segmenting the internal limiting membrane (ILM) and retinal pigment epithelium (RPE) or Bruch’s membrane when the RPE was not present, while Mishra [51] expanded on this, segmenting nine additional layers. The most recent study by Khateri et al. [53] proposed a “pathology aware” segmentation approach that identified severely vs non-severely (unaffected or mildly) affected zones, which guided the model to segment 5 layers, or one respectively. The earlier three studies all implemented Dijkstra’s graph search algorithm and U-Net variations to achieve segmentations [50–52, 61]. Alonso-Canerio and Kugleman [50, 52] enhanced their architecture with 50% dropout regularization, residual connections between first and third convolution blocks, and pooling variations. Additionally their latter study by Kugleman [52] implemented Monte-Carlo dropout for segmentation accuracy assessment, while Khateris group [53] used a similar method to automate and enhance their model training. The sample sizes varied across studies: 44 eyes from 22 patients [50], 197 patient eyes with 20 control eyes [51], 18 patients [52], and 259 patients [53]. Pixel-wise MAE ranges were 0.23–1.12, 0.77–4.73, and 0.51–1.66 for studies [50–52] respectively, while Khateri [53] assessed their performance using DICE, with their highest performance being 0.99.

Three studies [54–56] combined retina layer segmentation with en-face feature maps for atrophy segmentation. In Mishra’s first study [55], they employed an ensemble of U-Nets, each using specific OCT en-face feature maps (minimum, median mean, maximum intensity, standard deviation, kurtosis, skewness, gray-level entropy and thickness) to predict future atrophy, with FAF images as ground truth. Each U-Net provided probability maps, with the highest pixel-wise activation determining final categorization. In their later study [56], this group implemented three models: ReConNet, ReConNet-Ensemble, and ReConNet-Interval to predict atrophy progression. The first two both use a two-step recurrent approach, generating an initial 18 month or 12 month atrophy prediction from earlier time points (months 0, 6, and 12), and then reimplementing these images to refine segmentation in a final pass. ReConNet uses serial FAF images to generate a predicted future FAF image, while ReConNet-Ensemble focuses on using the same OCT based en-face feature maps as their previous work [55]. Their third model, ReConNet-Interval, predicts only new, incremental atrophy growth by masking out already atrophic regions in FAF images. All of these frameworks utilize 2D Conv-LSTM layers to capture longitudinal changes. Pfau et al. [54] similarly leveraged a deep learning pipeline where following retina layer segmentation, they segmented en face atrophic lesions to longitudinally track photoreceptor loss through proxy measures like EZ-zone loss. This was achieved using a Deeplabv3 model with 132 eyes across 66 patients. Mishra’s first study [55] included 264 eyes in 155 patients with baseline and 6-month imaging, and 237 eyes with up to a 12-month follow-up. Their more recent study[56] analyzed 141 eyes in 100 patients with imaging at initial, 6, 12, and 18 months including 71 eyes with OCT imaging at initial and 6-month follow up.

## Phenotyping

All three phenotyping studies identified through our search aim to better characterize the extent of physiological function in patients with ABCA4R, whether it be through automated grouping, or inferred measurements using efficient, objective means.

The first study by Muller et al. [57] utilized OCT data to predict three clinical measures: ERG groupings, visual impairment severity, and BCVA. They developed ensemble learning models combining k-Nearest Neighbor (KNN), random forest, support vector machines (SVM) with radial basis function (RBF) kernel, and eXtreme Gradient Boosting (XGBoost) for classification tasks, and KNN, kernel ridge regression, SVM with RBF kernel, and XGBoost for regression tasks. The study analyzed 156 subjects (45.5%, 35.3%, 19.2% in groups 1, 2, and 3 respectively by an expert Ophthalmologist) and 54 controls. For ERG prediction, OCT layer thickness data (feature set A) alone achieved prediction accuracies of 94.63%, 93.52%, and 96.99% categorizing patients into groups 1, 2, and 3. Visual impairment prediction achieved accuracies of 92.25%, 86.10%, 89.27%, and 88.64% for none, mild, moderate, and severe categories using feature set A. For BCVA prediction, feature set A and all structural data (feature set B) achieved 35.39% and 53.55% of predictions within 0.1 dB of ground truth respectively.

In a subsequent study, Muller and authors [58] utilized OCT data to predict MP response. Using a random forest regression, the authors achieved an overall MAE of 4.86 dB, with lower errors in peripheral measures (ETDRS 20–30 visual angle) compared to central measures (ETDRS 0–10 visual angle). The study tested five feature sets incorporating various clinical measures, with only the inclusion of initial MP measures showing significant improvement over OCT features alone.

In the final phenotyping study, Glinton and authors [59] focused on ERG phenotyping using ensemble models (SVM, Adaboost with decision trees, logistic regression). The model incorporated dark adapted (DA) 10, light adapted (LA) 3, LA 30 Hz flicker recordings, patient age, and pupil size data from 597 individuals (344, 44, and 209 patients in groups 1, 2, and 3). The model achieved 91.8% overall accuracy, with group-specific accuracies of 96.7%, 39.3%, and 93.8%. An alternative model classifying patients into group 1 or combined groups 2 & 3 achieved 93.6% accuracy, with 0.91 sensitivity, 0.95 specificity, and 0.93 mean AUC.

## Discussion

This scoping review covers recent ML applications in ABCA4R, with a focus on the tasks of image segmentation and phenotyping. The overall goal is to examine methodologies, innovations, and performance outcomes for key problems such as segmentation of flecks, atrophic lesions, retinal layers, the combination of these methods (mixed methods) as well as phenotyping. By exploring these studies, this review highlights challenges met in the field, innovations to address, and how they ultimately enhance our understanding of disease progression while improving clinical and research capabilities.

Atrophic lesion segmentation stands out as a key ML task for characterizing ABCA4R progression. The first attempt was by Wang et al. [45] who utilized a U-Net model with Adaptive Moment Estimation (Adam) [62] instead of the traditional Stochastic Gradient Descent (SGD). A unique aspect of this study was what data was used to train, validate, and test the models. In the segmentation task, the model was trained on 200 AMD subjects, validated on 20 AMD subjects, and tested on 100 subjects with AMD and ABCA4R yielding DICE’s of 0.94 and 0.87 respectively. Although both AMD and ABCA4R exhibit similar physical fleck lesions, the molecular mechanisms and demographic populations these occur in differ, possibly explaining this performance disparity. Nonetheless, for rare diseases like ABCA4R, implementing data from related pathologies with comparable manifestations can be an invaluable strategy to significantly improve DL model performance.

The same research group conducted a follow-up study using a U-Net architecture for atrophy segmentation, introducing an improved model with self-attention and soft-labels [46, 63]. The self-attention mechanism is embedded in the skip connections of the U-Net learning model, learning regions of images that are most relevant for the task, and distributes weights in a way that favors these regions during evaluation. As a result, information not contained in the self-attention component, like for example the periphery of the image, progressively receives lower weights, and the model learns to ignore this area. This study also implemented soft labels that provide a probability spectrum for each pixel rather than a binary true–false classification. A temperature parameter (T) controls the softness of these labels, with higher values representing lower levels of certainty, and vice versa, allowing models to handle noise and variation in their task rather than flat 0 and 1 for each pixel. The additions of self-attention and soft labels proved beneficial as the traditional U-Net and Self-attended U-Net obtained DICE values of 0.65 and 0.79 along with accuracies of 0.90 and 0.95 respectively. Another group Zhao et al. [47] also segmented atrophic lesion in FAF images, and refined a U-Net model to contain ResNet50 as the encoder. The novelty of this study involved a focus on the models ability to segment DDAF and QDAF atrophic lesions, which have demonstrated potential utility as outcome measures in clinical trials [27]. Their study achieved high DICE values of 0.90 for DDAF segmentation and 0.55 for QDAF. Even intraclass correlation between manual users for QDAF segmentation was poor, indicating this as a ubiquitously challenging task. Other studies have also demonstrated difficulty performing QDAF segmentation [64], highlighting it as a challenging task, but also potentially as a poorly defined one. Before further improvements in QDAF segmentation can be achieved, it may be necessary to further simplify these labels, like restricting segmentation problems to a specified radius, or including other thresholds to better classify QDAF groups, enabling DL models to find hidden patterns for improved performance within these subgroups.

Tracking and segmenting peripheral flecks has been of interest in ABCA4R for the purpose of phenotyping and prognostication, as these lesions can extend well past the macula, correlate with severity, and could serve as early quantifiable indicators of retinal pathology[65–67]. In studies segmenting flecks, they assessed these flecks with quantitative measures including ETDRS visual angles [48], and individual fleck metrics [49]. Specifically in [48], mean differences in fleck area (mm^2^) between manual graders and the DL model were − 0.03, 0.01, and − 0.03 for visual angles 0–10, 10–20, and 20–30 respectively. Given these results, it is possible during training the model developed in a manner that favored accurately segmenting flecks between visual angles 10–20, more so than flecks located in visual angles 0–10 and 20–30. Using models trained and tested specifically on particular regions of the fundus (0–10, 10–20, and 20–30) could result in tailored model development and ultimately better performance. Alternatively, Sabharwal et al.[49] used k-means clustering to reveal two distinct subtypes of flecks, with cluster 1 flecks being smaller, more circular, and located further from the fovea, while cluster 2 were larger, ovoid, and closer to the fovea. They also found a negative correlation between number of cluster 1 flecks and patient age. Future work could look to combine these metrics and longitudinally track these (and other) characteristics in order to better understand ABCA4R phenotypes.

In the task of retinal layer segmentation, Kugleman [50] and Alonso-Caneiro [52] implemented 50% dropout regularization, which works by randomly dropping half of the units within each layer of the model [68]. Here, units refer to the individual neurons within each layer of a neural network that apply weights to inputs to generate a new output. As a result, dropout prevents the model from relying on the same paths, and forces the network to develop a generalized model that avoids overfitting. Interestingly, this was the only DL model reported herein to average model weights for their final analysis. This was done due to the random initialization of weights, which inherently causes variability in performance. However, averaging weights can lead to learning paths that may not align logically with the goal output, as different initializations may favor different paths. An ensemble approach could be more robust, as it would combine predictions from models with different paths yet arrive at the same prediction.

Nonetheless, ensemble methods were successfully implemented in the mixed method and phenotyping studies. Ensemble learning methods refer to the aggregation of multiple individual models, each with their own strengths, in order to form a balanced final prediction. Mishra [55] formed an ensemble model where each individual U-Net was developed using a different OCT en-face feature map. Then for each pixel, whichever U-Net had the highest activation for that pixel space was used in the final image. By developing individual U-Net models for each en-face feature set, individual model performance for each feature could be evaluated. This helps address the inherent black-box issue in DL models where it is difficult to understand which features contribute to successful model development and execution [69]. Additionally, different feature maps that may be particularly better suited for segmenting different regions, layers, and or stages of disease can be implemented. When categorizing ERG, Glinton and co-authors [59] also used an ensemble model, which included SVM, Adaboost with decision trees, and logistic regression. Though ERG groupings 1, 2, and 3 are based on macular dysfunction, scotopic vision, and photopic vision respectively, they report a phenotypic spectrum of ERG responses in those with ABCA4R more so than distinctive classifications. For example, group 2 typically displays only differences in scotopic responses, however small, yet discernable differences can be made in photopic responses between it and groups 1 and 3. When refining decision trees during model development, Adaboost works by placing higher weights on examples that were incorrectly classified. Future rounds of model training then focus more of their attention on cases where weights are higher, and iteratively find a balance that prioritizes edge cases, while yielding a reasonable accuracy.

Muller and colleagues similarly followed, developing ensemble models for both classification and regression tasks in their two studies [57, 58]. They found that the smallest feature set composed of only OCT thickness data performed no worse than other feature sets composed of additional measures like false-positive responses, mean reaction time during MP recording, demographic data, ERG groupings, age of onset, BCVA, and previous MP measures. The exception to this was the inclusion of foveal involvement as measured by OCT for ‘inferred BCVA’ and previous MP measures strengthened prediction of future MP recordings, or ‘inferred retinal sensitivity’. OCT offers a direct high resolution quantification of retinal structure—any additional features that do not confer a direct and or higher resolution for the measure of interest will likely not improve performance. Given ensembling is a computationally intensive approach than any single model on its own, features with lower relevance and or resolution can be removed to limit resources.

A unique application of retinal layer segmentation was used to characterize ABCA4 variant severity based on ellipsoid zone (EZ) metrics. Pfau and colleagues [54] introduced an “EZ-loss age” measure derived from longitudinal spectral domain OCT scans, where they first measure the area of EZ loss across multiple visits, apply a square-root transformation to create a linearized metric, and fit a linear model over time. By correlating this with two ABCA4 alleles that each patient carries, this study quantified allele severity. For instance, the predicted missense change p.Gly1961Glu was repeatedly linked to later-onset and slow progression, while variants like c.5461-10T > C behaved similarly to null alleles, predicting earlier onset and faster disease course. Additionally, other alleles like p.Cys2150Tyr appeared to correlate with even more severe clinical manifestations than null alelles. From this, clinicians may be better able to highlight differences among ABCA4 variants that traditional clinical measurements may underestimate. Importantly, this study demonstrates the feasibility of a machine learning based segmentation that enables scalable, objective quantification of measures like EZ loss for genotype–phenotype correlations, and informing gene-targeted interventions.

Khateri et al. [53] proposed a “pathology-aware” segmentation framework for OCT that used a box-detector to isolate severely affected regions, following U-Net sublayer segmentation in healthier areas. Regions of the retina with severe degeneration can exhibit loss of multiple retina layers, influencing segmentation paths, and degrading performance. By automatically excluding regions with extensive damage, this approach segments 1 layer as the retina within this region, while continuing to segment five sublayers outside this region. This group also sampled B-scans from the center and periphery, and ensured denser sampling of central B-scans, as this is where pathology typically arises. Lastly, the group implemented an automated, iterative learning pipeline that leveraged a Monte Carlo Dropout method [70]. In Monte Carlo iterative learning, the model is first trained on a small labeled dataset, termed the “seed”. The model, trained on the seed dataset is then used to make predictions on an unlabeled pool. The model performs multiple predictions of unlabeled samples—samples with high variability across predictions are considered to have “high uncertainty”, prompting human graders to label them, and are then processed back into the model for learning. Samples that exhibit high uncertainty are assumed to be the most “informative” to label next. This process continues to loop through all samples iteratively assessing uncertainty until model performance stagnates. This ultimately conserves resources by having human graders create ground truth labels only for samples that would improve model performance and generalizability. Overall, this study demonstrates an impressive collection of data considerations and automation methods that help address the multifaceted imaging and ML challenges specific to ABCA4R.

## Conclusion

This scoping review focused on surveying the current landscape of Machine Learning applications in ABCA4R, specifically image segmentation and phenotyping. Over the course of the timeframe (2018–2025) involved in this scoping review, there has been a clear progression in methods like the use of en-face feature maps for segmentation tasks and atrophy prediction, or the utilization of monte-carlo dropout to improve development pipelines. In light of current clinical trials, there is a need to develop quick and objective proxies for other related measures that have an established biological relationship. Development of quantitative, objective metrics that can be automated would reduce work by clinicians and scientists while also standardizing methods. These advancements would also benefit clinical settings where patients can receive comprehensive yet concise testing, allocating more time for other parts of their care.

## Data Availability

No datasets were generated or analysed during the current study.

## References

[1] Stargardt K (1909) Über familiäre, progressive degeneration in der Maculagegend des Auges. Arbeitsphysiologie 71:534–550

[2] Molday LL, Rabin AR, Molday RS (2000) ABCR expression in foveal cone photoreceptors and its role in Stargardt macular dystrophy. Nat Genet 25:257–258 10888868 10.1038/77004

[3] Allikmets R, Singh N, Sun H et al (1997) A photoreceptor cell-specific ATP-binding transporter gene (ABCR) is mutated in recessive Stargardt macular dystrophy. Nat Genet 15:236–246 9054934 10.1038/ng0397-236

[4] Lenis TL, Hu J, Ng SY et al (2018) Expression of ABCA4 in the retinal pigment epithelium and its implications for Stargardt macular degeneration. Proc Natl Acad Sci USA 115:E11120–E11127 30397118 10.1073/pnas.1802519115 PMC6255167

[5] Mizobuchi K, Hayashi T, Tanaka K et al (2024) Genetic and clinical features of ABCA4-associated retinopathy in a Japanese nationwide cohort. Am J Ophthalmol 264:36–43 38499139 10.1016/j.ajo.2024.03.007

[6] Pontikos N, Arno G, Jurkute N et al (2020) Genetic basis of inherited retinal disease in a molecularly characterized cohort of more than 3000 families from the United Kingdom. Ophthalmology 127:1384–1394 32423767 10.1016/j.ophtha.2020.04.008 PMC7520514

[7] Goetz KE, Reeves MJ, Gagadam S et al (2020) Genetic testing for inherited eye conditions in over 6,000 individuals through the eyeGENE network. Am J Med Genet C Semin Med Genet 184:828–837 32893963 10.1002/ajmg.c.31843 PMC8162059

[8] Tanna P, Strauss RW, Fujinami K, Michaelides M (2017) Stargardt disease: clinical features, molecular genetics, animal models and therapeutic options. Br J Ophthalmol 101:25–30 27491360 10.1136/bjophthalmol-2016-308823 PMC5256119

[9] Strauss RW, Ho A, Muñoz B et al (2016) The natural history of the progression of atrophy secondary to Stargardt disease (ProgStar) studies: design and baseline characteristics: ProgStar report no. 1. Ophthalmology 123:817–828 26786511 10.1016/j.ophtha.2015.12.009

[10] Tsang SH, Sharma T (2018) Stargardt disease. Adv Exp Med Biol 1085:139–151 30578500 10.1007/978-3-319-95046-4_27

[11] Kohli P, Tripathy K, Kaur K (2024) Stargardt Disease. In: StatPearls. StatPearls Publishing, Treasure Island (FL) 36508525

[12] Clinicaltrials.gov. https://clinicaltrials.gov/. Accessed 22 Feb 2025

[13] Clinicaltrials.gov. https://clinicaltrials.gov/study/NCT05417126?cond=stargardt%20disease&rank=9. Accessed 1 Mar 2025

[14] Clinicaltrials.gov. https://clinicaltrials.gov/study/NCT03772665?cond=stargardt%20disease&rank=8. Accessed 1 Mar 2025

[15] Cideciyan AV, Swider M, Aleman TS et al (2009) ABCA4 disease progression and a proposed strategy for gene therapy. Hum Mol Genet 18:931–941 19074458 10.1093/hmg/ddn421 PMC2640207

[16] Fujinami K, Lois N, Davidson AE et al (2013) A longitudinal study of Stargardt disease: clinical and electrophysiologic assessment, progression, and genotype correlations. Am J Ophthalmol 155:1075-1088.e13 23499370 10.1016/j.ajo.2013.01.018

[17] Rotenstreich Y, Fishman GA, Anderson RJ (2003) Visual acuity loss and clinical observations in a large series of patients with Stargardt disease. Ophthalmology 110:1151–1158 12799240 10.1016/S0161-6420(03)00333-6

[18] Miraldi Utz V, Coussa RG, Marino MJ et al (2014) Predictors of visual acuity and genotype-phenotype correlates in a cohort of patients with Stargardt disease. Br J Ophthalmol 98:513–518 24457364 10.1136/bjophthalmol-2013-304270

[19] Pfau M, Huryn LA, Boyle MP et al (2023) Natural history of visual dysfunction in ABCA4 retinopathy and its genetic correlates. Am J Ophthalmol 253:224–232 37211138 10.1016/j.ajo.2023.05.014 PMC10524499

[20] Kong X, Strauss RW, Michaelides M et al (2016) Visual acuity loss and associated risk factors in the retrospective progression of Stargardt disease study (ProgStar report no. 2). Ophthalmology 123:1887–1897 27378015 10.1016/j.ophtha.2016.05.027

[21] Schönbach EM, Ibrahim MA, Strauss RW et al (2017) Fixation location and stability using the MP-1 microperimeter in Stargardt disease: ProgStar report no. 3. Ophthalmol Retina 1:68–76 31047397 10.1016/j.oret.2016.08.009

[22] Kong X, West SK, Strauss RW et al (2017) Progression of visual acuity and fundus autofluorescence in recent-onset Stargardt disease: ProgStar study report #4. Ophthalmol Retina 1:514–523 31047445 10.1016/j.oret.2017.02.008

[23] Strauss RW, Muñoz B, Ho A et al (2017) Incidence of atrophic lesions in Stargardt disease in the progression of atrophy secondary to Stargardt disease (ProgStar) study: report no. 5. JAMA Ophthalmol 135:687–695 28542697 10.1001/jamaophthalmol.2017.1121 PMC5710205

[24] Kong X, Strauss RW, Cideciyan AV et al (2017) Visual acuity change over 12 months in the prospective progression of atrophy secondary to Stargardt disease (ProgStar) study: ProgStar report number 6. Ophthalmology 124:1640–1651 28549516 10.1016/j.ophtha.2017.04.026

[25] Schönbach EM, Wolfson Y, Strauss RW et al (2017) Macular sensitivity measured with microperimetry in Stargardt disease in the Progression of Atrophy Secondary to Stargardt Disease (ProgStar) study: report no. 7: report no. 7. JAMA Ophthalmol 135:696–703 28542693 10.1001/jamaophthalmol.2017.1162 PMC6584711

[26] Fujinami K, Strauss RW, Chiang JP-W et al (2019) Detailed genetic characteristics of an international large cohort of patients with Stargardt disease: ProgStar study report 8. Br J Ophthalmol 103:390–397 29925512 10.1136/bjophthalmol-2018-312064 PMC6579578

[27] Strauss RW, Muñoz B, Ho A et al (2017) Progression of Stargardt disease as determined by fundus autofluorescence in the retrospective progression of Stargardt disease study (ProgStar report no. 9). JAMA Ophthalmol 135:1232–1241 29049437 10.1001/jamaophthalmol.2017.4152 PMC5710470

[28] Kong X, Fujinami K, Strauss RW et al (2018) Visual acuity change over 24 months and its association with foveal phenotype and genotype in individuals with Stargardt disease: ProgStar study report no. 10. JAMA Ophthalmol 136:920–928 29902293 10.1001/jamaophthalmol.2018.2198 PMC6142940

[29] Strauss RW, Kong X, Ho A et al (2019) Progression of Stargardt Disease as determined by fundus autofluorescence over a 12-month period: ProgStar report no. 11: ProgStar report no. 11. JAMA Ophthalmol 137:1134–1145 31369039 10.1001/jamaophthalmol.2019.2885 PMC6681653

[30] Schönbach EM, Strauss RW, Kong X et al (2018) Longitudinal changes of fixation location and stability within 12 months in Stargardt disease: ProgStar report no. 12. Am J Ophthalmol 193:54–61 29890160 10.1016/j.ajo.2018.06.003 PMC7083180

[31] Schönbach EM, Strauss RW, Muñoz B et al (2020) Longitudinal microperimetric changes of macular sensitivity in Stargardt Disease after 12 months: ProgStar report no. 13: ProgStar report no. 13. JAMA Ophthalmol 138:772–779 32463436 10.1001/jamaophthalmol.2020.1735 PMC7256863

[32] Schönbach EM, Strauss RW, Ibrahim MA et al (2020) Faster sensitivity loss around dense scotomas than for overall macular sensitivity in Stargardt disease: ProgStar report no. 14. Am J Ophthalmol 216:219–225 32222369 10.1016/j.ajo.2020.03.020

[33] Schönbach EM, Janeschitz-Kriegl L, Strauss RW et al (2021) The progression of Stargardt disease using volumetric hill of vision analyses over 24 months: ProgStar report no.15. Am J Ophthalmol 230:123–133 33951446 10.1016/j.ajo.2021.04.015

[34] Schönbach EM, Strauss RW, Cattaneo MEGV et al (2022) Longitudinal changes of fixation stability and location within 24 months in Stargardt disease: ProgStar report no. 16. Am J Ophthalmol 233:78–89 34298008 10.1016/j.ajo.2021.07.013

[35] Strauss RW, Ho A, Jha A et al (2023) Progression of Stargardt disease as determined by fundus autofluorescence over a 24-month period (ProgStar report no. 17). Am J Ophthalmol 250:157–170 36764427 10.1016/j.ajo.2023.02.003

[36] Strauss RW, Lang L, Ho A et al (2024) The progression of Stargardt disease as determined by spectral-domain optical coherence tomography over a 24-month period (ProgStar report no. 18). Ophthalmic Res 67:435–447 39004077 10.1159/000540028

[37] Aumann S, Donner S, Fischer J, Müller F (2019) Optical coherence tomography (OCT): principle and technical realization. In: Bille JF (ed) High resolution imaging in microscopy and ophthalmology: new frontiers in biomedical optics. Springer, Cham 32091677

[38] Frampton GK, Kalita N, Payne L et al (2017) Fundus autofluorescence imaging: systematic review of test accuracy for the diagnosis and monitoring of retinal conditions. Eye 31:995–1007 28282065 10.1038/eye.2017.19 PMC5519265

[39] McCulloch DL, Marmor MF, Brigell MG et al (2015) ISCEV standard for full-field clinical electroretinography (2015 update). Doc Ophthalmol 130:1–12 25502644 10.1007/s10633-014-9473-7

[40] Lois N, Holder GE, Bunce C et al (2001) Phenotypic subtypes of Stargardt macular dystrophy-fundus flavimaculatus. Arch Ophthalmol 119:359–369 11231769 10.1001/archopht.119.3.359

[41] Jin K, Ye J (2022) Artificial intelligence and deep learning in ophthalmology: current status and future perspectives. Adv Ophthalmol Pract Res 2:100078 37846285 10.1016/j.aopr.2022.100078 PMC10577833

[42] Li Z, Wang L, Wu X et al (2023) Artificial intelligence in ophthalmology: the path to the real-world clinic. Cell Rep Med 4:101095 37385253 10.1016/j.xcrm.2023.101095 PMC10394169

[43] Page MJ, McKenzie JE, Bossuyt PM et al (2021) The PRISMA 2020 statement: an updated guideline for reporting systematic reviews. BMJ 372:n71 33782057 10.1136/bmj.n71 PMC8005924

[44] Stephan R (2022) Mondo: unifying diseases for the world, by the world. medRxiv. 10.1101/2022.04.13.22273750

[45] Wang Z, Sadda SR, Hu Z (2019) Deep learning for automated screening and semantic segmentation of age-related and juvenile atrophic macular degeneration

[46] Wang Z, Sadda SR, Lee A, Hu ZJ (2022) Automated segmentation and feature discovery of age-related macular degeneration and Stargardt disease via self-attended neural networks. Sci Rep 12:14565 36028647 10.1038/s41598-022-18785-6 PMC9418226

[47] Zhao PY, Branham K, Schlegel D et al (2022) Automated segmentation of autofluorescence lesions in Stargardt disease. Ophthalmol Retina 6:1098–1104 35644472 10.1016/j.oret.2022.05.020 PMC10370158

[48] Charng J, Xiao D, Mehdizadeh M et al (2020) Deep learning segmentation of hyperautofluorescent fleck lesions in Stargardt disease. Sci Rep 10:16491 33020556 10.1038/s41598-020-73339-y PMC7536408

[49] Sabharwal J, Liu TYA, Antonio-Aguirre B et al (2024) Automated identification of fleck lesions in Stargardt disease using deep learning enhances lesion detection sensitivity and enables morphometric analysis of flecks. Br J Ophthalmol 108:1226–1233 38408857 10.1136/bjo-2023-323592

[50] Kugelman J, Alonso-Caneiro D, Chen Y et al (2020) Retinal boundary segmentation in Stargardt disease optical coherence tomography images using automated deep learning. Transl Vis Sci Technol 9:12 33133774 10.1167/tvst.9.11.12 PMC7581491

[51] Mishra Z, Wang Z, Sadda SR, Hu Z (2021) Automatic segmentation in multiple OCT layers for Stargardt disease characterization via deep learning. Transl Vis Sci Technol 10:24 34004000 10.1167/tvst.10.4.24 PMC8083069

[52] Alonso-Caneiro D, Kugelman J, Tong J, et al (2021) Use of uncertainty quantification as a surrogate for layer segmentation error in Stargardt disease retinal OCT images

[53] Khateri P, Koottungal T, Wong D et al (2025) Looking outside the box with a pathology aware AI approach for analyzing OCT retinal images in Stargardt disease. Sci Rep 15:4739 39922894 10.1038/s41598-025-85213-w PMC11807158

[54] Pfau M, Cukras CA, Huryn LA et al (2022) Photoreceptor degeneration in ABCA4-associated retinopathy and its genetic correlates. JCI Insight. 10.1172/jci.insight.155373 35076026 10.1172/jci.insight.155373 PMC8855828

[55] Mishra Z, Wang Z, Sadda SR, Hu Z (2023) Using ensemble OCT-derived features beyond intensity features for enhanced Stargardt atrophy prediction with deep learning. Appl Sci (Basel). 10.3390/app13148555 39086558 10.3390/app13148555 PMC11288976

[56] Mishra Z, Wang ZC, Xu E et al (2024) Recurrent and concurrent prediction of longitudinal progression of Stargardt atrophy and geographic atrophy towards comparative performance on optical coherence tomography as on fundus autofluorescence. Appl Sci. 10.3390/app14177773

[57] Müller PL, Treis T, Odainic A et al (2020) Prediction of function in ABCA4-related retinopathy using ensemble machine learning. J Clin Med. 10.3390/jcm9082428 32751377 10.3390/jcm9082428 PMC7463567

[58] Müller PL, Odainic A, Treis T et al (2021) Inferred retinal sensitivity in recessive Stargardt disease using machine learning. Sci Rep 11:1466 33446864 10.1038/s41598-020-80766-4 PMC7809282

[59] Glinton SL, Calcagni A, Lilaonitkul W et al (2022) Phenotyping of *ABCA4* retinopathy by machine learning analysis of full-field electroretinography. Transl Vis Sci Technol 11:34 36178783 10.1167/tvst.11.9.34 PMC9527330

[60] Esengönül M, Marta A, Beirão J et al (2022) A systematic review of artificial intelligence applications used for inherited retinal disease management. Medicina (Kaunas) 58:504 35454342 10.3390/medicina58040504 PMC9028098

[61] Dijkstra EW (1959) A note on two problems in connexion with graphs. Numer Math (Heidelb) 1:269–271

[62] Kingma DP, Ba J (2014) Adam: a method for stochastic optimization. arXiv [cs.LG]

[63] Nguyen Q, Valizadegan H, Hauskrecht M (2014) Learning classification models with soft-label information. J Am Med Inform Assoc 21:501–508 24259520 10.1136/amiajnl-2013-001964 PMC3994863

[64] Kuehlewein L, Hariri AH, Ho A et al (2016) Comparison of manual and semiautomated fundus autofluorescence analysis of macular atrophy in Stargardt disease phenotype. Retina 36:1216–1221 26583307 10.1097/IAE.0000000000000870

[65] Ervin A-M, Strauss RW, Ahmed MI et al (2019) A workshop on measuring the progression of atrophy secondary to stargardt disease in the progstar studies: Findings and lessons learned. Transl Vis Sci Technol. 10.1167/tvst.8.2.16 31019847 10.1167/tvst.8.2.16 PMC6469878

[66] Bax NM, Lambertus S, Cremers FPM et al (2019) The absence of fundus abnormalities in Stargardt disease. Arbeitsphysiologie 257:1147–1157 10.1007/s00417-019-04280-8 30903310

[67] Chen L, Lee W, de Carvalho Jr JRL et al (2019) Multi-platform imaging in ABCA4-associated disease. Sci Rep 9:6436 31015497 10.1038/s41598-019-42772-z PMC6478712

[68] Srivastava N, Hinton GE, Krizhevsky A et al (2014) Dropout: a simple way to prevent neural networks from overfitting. J Mach Learn Res 15:1929–1958

[69] London AJ (2019) Artificial intelligence and black-box medical decisions: accuracy versus explainability. Hastings Cent Rep 49:15–21 30790315 10.1002/hast.973

[70] Blok PM, Kootstra G, Elghor HE et al (2022) Active learning with MaskAL reduces annotation effort for training Mask R-CNN on a broccoli dataset with visually similar classes. Comput Electron Agric 197:106917

